# The Arabidopsis WRINKLED1 transcription factor affects auxin homeostasis in roots

**DOI:** 10.1093/jxb/erx275

**Published:** 2017-08-26

**Authors:** Que Kong, Wei Ma, Haibing Yang, Guojie Ma, Jenny J Mantyla, Christoph Benning

**Affiliations:** 1 MSU-DOE Plant Research Laboratory, Michigan State University, East Lansing, MI, USA; 2 Department of Biochemistry and Molecular Biology, Michigan State University, East Lansing, MI, USA; 3 Great Lakes Bioenergy Research Center, Michigan State University, East Lansing, MI, USA; 4 Department of Plant Biology, Michigan State University, East Lansing, MI, USA; 5 Department of Horticulture and Landscape Architecture, Purdue University, West Lafayette, IN, USA

**Keywords:** Arabidopsis, auxin, DNA-binding motif, gene expression, transcription factor, root development

## Abstract

WRINKLED1 (WRI1) is a key transcriptional regulator of fatty acid biosynthesis genes in diverse oil-containing tissues. Loss of function of Arabidopsis *WRI1* leads to a reduction in the expression of genes for fatty acid biosynthesis and glycolysis, and concomitant strong reduction of seed oil content. The *wri1-1* loss-of-function mutant shows reduced primary root growth and decreased acidification of the growth medium. The content of a conjugated form of the plant growth hormone auxin, indole-3-acetic acid (IAA)-Asp, was higher in *wri1-1* plants compared with the wild-type. *GH3.3*, a gene encoding an enzyme involved in auxin degradation, displayed higher expression in the *wri1-1* mutant. EMSAs demonstrated that AtWRI1 bound to the promoter of *GH3.3*. Specific AtWRI1-binding motifs were identified in the promoter of *GH3.3*. In addition, *wri1-1* displayed decreased auxin transport. Expression of some *PIN* genes, which encode IAA carrier proteins, was reduced in *wri1-1* plants as well. Correspondingly, AtWRI1 bound to the promoter regions of some *PIN* genes. It is well known that auxin exerts its maximum effects at a specific, optimal concentration in roots requiring a finely balanced auxin homeostasis. This process appears to be disrupted when the expression of *WRI1* and in turn a subset of its target genes are misregulated, highlighting a role for *WRI1* in root auxin homeostasis.

## Teaser

WRINKLED1 (WRI1) has targets other than fatty acid biosynthesis genes that mediate the growth of roots.

## Introduction

The growth regulator auxin (indole-3-acetic acid; IAA) plays an important role in plant morphogenesis and development such as growth of the shoot and root, hypocotyl elongation, etc. (Zhao *et al.*, 2001; Takase *et al.*, 2004; Woodward and Bartel, 2005). As part of auxin-mediated signaling pathways, expression of numerous genes is affected by tissue auxin concentration (Wang *et al.*, 2010). Auxin signaling marker genes (auxin-responsive genes) have been classified into three major categories: *Aux/IAA* genes, *SAUR* genes (small, auxin-induced RNA), and *GH3* genes (Ulmasov *et al.*, 1995; Woodward and Bartel, 2005; Khan and Stone, 2007; Zhang *et al.*, 2007).

Expression of *GH3* genes is generally associated with plant development (e.g. root and hypocotyl growth). Nakazawa *et al.* (2001) found that Arabidopsis *DFL1* (*GH3.6*) affects plant morphogenesis including the development of shoot, root, and hypocotyl (Nakazawa *et al.*, 2001). *GH3.9* was found to affect the growth of the primary root, and Arabidopsis (*Arabidopsis thaliana*) *gh3.9* (a *GH3.9* loss-of-function mutant) displays an elongated root compared with wild-type (WT) seedlings (Tatematsu *et al.*, 2008). Arabidopsis GH3.5 (WES1) was found to mediate auxin homeostasis, and overexpression of *GH3.5* led to reduced IAA levels, reduced plant growth, and disturbed adventitious root development (Park *et al.*, 2007). *GH3.2* (*YDK1*) overexpression in transgenic Arabidopsis plants also displayed reduced primary root growth and short hypocotyls (Takase *et al.*, 2004).

Recently, several *GH3* genes were found to encode enzymes which form IAA-amino acid conjugates (Staswick *et al.*, 2002, 2005). Mutations of two *GH3* genes in *Physcomitrella patens* led to elevated free auxin content and enhanced sensitivity to auxin application (Ludwig-Muller *et al.*, 2009). This evidence suggests that GH3 proteins play a role in balancing auxin pools, which also supports the notion that the role of GH3 proteins is evolutionarily conserved in mediating auxin homeostasis (Chapman and Estelle, 2009).

*GH3* genes have also been studied with regard to their transcriptional regulation by promoter analysis and identification of the transcription factors responsible. The majority of *GH3* genes contain auxin-responsive elements (AuxREs) and other *cis*-elements associated with stresses and other hormone responses (Woodward and Bartel, 2005; Ostrowski and Jakubowska, 2013). *DFL1* (*GH3.6*), *AtGH3a*, and *GH3.17*, but not *GH3.3*, are the targets of the transcription factor ARF8 (Tian *et al.*, 2004). The Arabidopsis MYB77 transcription factor affects expression of *GH3.2* and *GH3.3* (Shin *et al.*, 2007). The tobacco bZIP transcription factor NtBZI-1 binds to a G-box-related element (GRE) in the promoter region of *NtGH3* which affects the expression of *NtGH3* (Heinekamp *et al.*, 2004).

Members of the PIN (PIN-FORMED) proteins have been known to play an important role in auxin transport (Galweiler *et al.*, 1998; Luschnig *et al.*, 1998; Zazimalova *et al.*, 2010). Both environmental cues and plant hormones are found to be crucial factors in *PIN* expression (Peer *et al.*, 2004; Kimbrough *et al.*, 2005; Vieten *et al.*, 2005; Keuskamp *et al.*, 2010; Sassi *et al.*, 2012). Although *PIN* expression has been investigated broadly (Aida *et al.*, 2004; Vieten *et al.*, 2005), not much is known regarding the transcription factors which directly control the expression of *PIN* genes. Recently, an Arabidopsis MADS transcription factor, XAL2, has been identified to mediate auxin transport by regulation of *PIN1* and *PIN4* (Garay-Arroyo *et al.*, 2013). The Arabidopsis MYB88 transcription factor was recently found to control the expression of *PIN3* and *PIN7* directly (Wang *et al.*, 2015). The Arabidopsis transcription factors IDD14 and IDD16 have been found to activate the expression of *PIN1* (Cui *et al.*, 2013).

Arabidopsis WRINKLED1 (WRI1) is an APETALA2 (AP2) transcription factor (Cernac and Benning, 2004; Masaki *et al.*, 2005). The *wri1-1* mutant displays an ~80% reduction of seed oil content (Focks and Benning, 1998) and decreased expression of genes encoding fatty acid and glycolytic enzymes (Ruuska *et al.*, 2002). Overexpression of *AtWRI1* led to increased expression of *de novo* fatty acid biosynthesis genes (Sanjaya *et al.*, 2011) and increased oil content in seeds and leaves of transgenic Arabidopsis plants (Cernac and Benning, 2004; Sanjaya *et al.*, 2011). The AW-box has been characterized as an AtWRI1-binding motif which is conserved in promoter regions of AtWRI1 target fatty acid biosynthesis genes (Maeo *et al.*, 2009). *WRI1* orthologs have been found to affect expression of *de novo* fatty acid biosynthesis genes and oil biosynthesis in different plant species (Liu *et al.*, 2010; Shen *et al.*, 2010; Pouvreau *et al.*, 2011; Ma *et al.*, 2013; Wu *et al.*, 2014; Yang *et al.*, 2015). Transient expression of *AtWRI1* and *WRI1* orthologs in tobacco leaves led to oil production (Vanhercke *et al.*, 2013; Grimberg *et al.*, 2015; Ma *et al.*, 2015). AtWRI1 is targeted by the 26S proteasome-mediated degradation pathway (Chen *et al.*, 2013), and the PEST motif in the C-terminal intrinsically disordered region (IDR) of AtWRI1 affects the stability of AtWRI1 and oil production (Ma *et al.*, 2015). 14-3-3 proteins were recently found to interact with AtWRI1 and affect AtWRI1 transcriptional activity and oil biosynthesis (Ma *et al.*, 2016).

In this study, we describe that Arabidopsis *wri1-1* plants display altered development of the primary root. Auxin content and the expression of *GH3* genes are affected in *wri1-1*. Notably, we found that AtWRI1 binds to the promoter of *GH3.3* (_*pro*_*GH3.3*). We identified a putative binding element for AtWRI1 located in _*pro*_*GH3.3* different from the known *cis*-element (AW-box) (Maeo *et al.*, 2009).

## Materials and methods

### Plant materials

Plant materials and growth conditions have been previously described (Ma *et al.*, 2013, 2015). Transgenic *wri1-1* lines overexpressing an *AtWRI1-TAP* (line #6-5 and #7-2) were previously described (Ma *et al.*, 2013). Seed sterilization followed the method described previously (Ma *et al.*, 2013).

### Growth medium pH assay

The pattern of pH change in the growth medium around the roots of Arabidopsis seedlings was determined as previously published (Ma *et al.*, 2006; Yang *et al.*, 2007). Arabidopsis seedlings were first grown vertically on normal medium for 3 d before being transferred to growth medium containing the pH indicator, bromocresol purple. Images were taken 7 d after the seedlings were transferred to pH indicator growth medium.

### Quantification of IAA and IAA-Asp in Arabidopsis

One-week-old Arabidopsis seedlings grown vertically on normal medium were harvested (~100 mg FW per sample) and immediately frozen in liquid nitrogen. Samples were sent to the Danforth Center Proteomics & Mass Spectrometry core facility for acidic plant hormone analysis. The detailed protocol can be found at their website (http://www.danforthcenter.org/docs/default-source/CoreFacilities/pmsf/plant-hormone-analysis_customer-instructions_2014.pdf?sfvrsn=0, last accessed 27 July 2017).

### Protein expression and EMSA

AtWRI1^58–240^ was subcloned into the pET41a-6×His vector. The primers used for subcloning AtWRI1^58–240^ were 5'-GCAGGATCC GCTTCTACCCGA-3' (forward) and 5'-TAACTCGAGTTACG GGAAAACACC-3' (reverse). Protein induction, extraction, and purification were performed as previously published (Kong *et al.*, 2012; Ma *et al.*, 2015). EMSAs were performed according to Kong *et al.* (2012).

### RNA extraction and quantitative real-time PCR

Methods for RNA extraction, cDNA synthesis, and quantitative real-time PCR (qRT-PCR) were as previously described (Ma *et al.*, 2015). The gene expression level was normalized to an internal standard, the *Protein Phosphatase 2A* (*PP2A*) gene. The primers used for qRT-PCR are described in Supplementary Table S1 at *JXB* online. The primers used for *PP2A* were previously published (Czechowski *et al.*, 2005). The primers used for *GH3.2*, *GH3.3*, *GH3.4*, *GH3.5*, *GH3.6*, and *GH3.7* were according to González-Lamothe *et al.* (2012), and the primers for *ARF6*, *ARF8*, and *ARF17* were according to Gutierrez *et al.* (2009).

### [^3^H]IAA transport assays

Ten-day-old seedlings were used for [^3^H]IAA transport assays. The assays were as described in Blakeslee *et al.* (2007).

## Results

### *AtWRI1* affects Arabidopsis root development

**Fig. 1.**
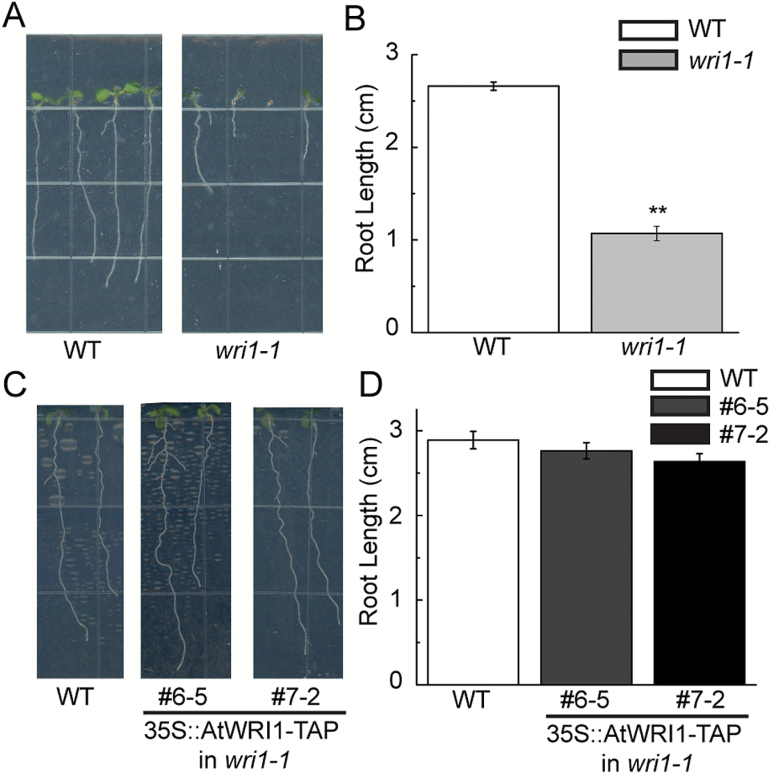
Primary root length of wild-type (WT), *wri1-1*, an *AtWRI1* loss-of-function mutant, and transgenic *wri1-1* plants overexpressing *AtWRI1-TAP* (lines 6-5 and 7-2). Root growth (A) and root length measurement (B) of 1-week-old seedlings of the WT and *wri1-1*. Primary root length was typically measured on 36 seedlings to calculate a mean value. Results are shown as means ±SE (*n*=36). Root growth (C) and root length measurement (D) of 10-day-old seedlings of the WT and transgenic *wri1-1* overexpressing *AtWRI1-TAP* (lines 6-5 and 7-2). Primary root length was typically measured on 31 seedlings for the WT or 22 seedlings for lines 6-5 and 7-2 to calculate a mean value. Results are shown as means ±SE (*n*=31 for the WT and *n*=22 for lines 6-5 and 7-2). Primary root length of the *wri1-1* mutant displayed statistically significant differences compared with the WT (*P*<0.01, *t*-test), as indicated by **. (This figure is available in colour at *JXB* online.)

The *AtWRI1* transcript is most abundant in the embryo and has generally low abundance in vegetative tissues such as leaves (Cernac and Benning, 2004). However, it should be noted that *AtWRI1* has high expression in tissues such as ovaries and roots, as determined by microarray analysis (Supplementary Fig. S1). Northern blots also detected *AtWRI1* at high levels in roots and flowers (Cernac and Benning, 2004). However, there is currently no report regarding *wri1*-related mutant phenotypes in either Arabidopsis ovaries or roots. Here, we observed the root morphology of the *wri1-1* mutant and determined a possible *AtWRI1* function in roots. The *wri1-1* mutant displayed reduced root length compared with WT seedlings (Fig. 1A, B). In order to test whether the root phenotype in the *wri1-1* mutant was due to the mutation of *AtWRI1*, root length in *wri1-1* transgenic plants overexpressing *AtWRI1-TAP* in lines #6-5 and #7-2 (Ma *et al.*, 2013) was determined to test for restoration of this phenotype. As shown in Fig. 1C and D, expression of *AtWRI1-TAP* restored the root growth in the *wri1-1* mutant.

Previous studies indicated that proton (H^+^) efflux is correlated with root growth. For example, Arabidopsis mutants or transgenic plants with increased root growth have been found to show enhanced H^+^ efflux (Ma *et al.*, 2006; Yang *et al.*, 2007). Hence, we investigated the H^+^ efflux out of *wri1-1* mutant roots employing a medium acidification assay. As shown in Supplementary Fig. S2, *wri1-1* roots displayed decreased H^+^ efflux.

### The *wri1-1* mutant accumulates an auxin metabolite

**Fig. 2.**
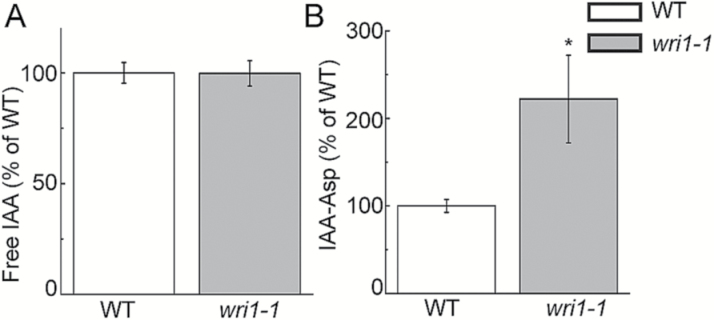
Quantification of IAA and IAA-Asp in WT and *wri1-1* plants by LC-MS/MS (1-week-old seedlings). Results are shown as means ±SE (*n*=4–5). * indicates a significant difference (*P*<0.05, *t*-test) from the WT.

Suspecting that auxin homeostasis was altered, we quantified IAA metabolites (free IAA and IAA-Asp) using LC-MS/MS. These IAA metabolites have been shown to be associated with Arabidopsis root growth (Quint *et al.*, 2009; Ludwig-Muller, 2011; Pencik *et al.*, 2013). Our result indicated that free IAA was not altered in the *wri1-1* mutant compared with the WT (Fig. 2A). However, tissue levels of IAA-Asp were higher in *wri1-1* (Fig. 2B).

### Expression of *GH3* genes is altered in the *wri1-1* mutant

**Fig. 3.**
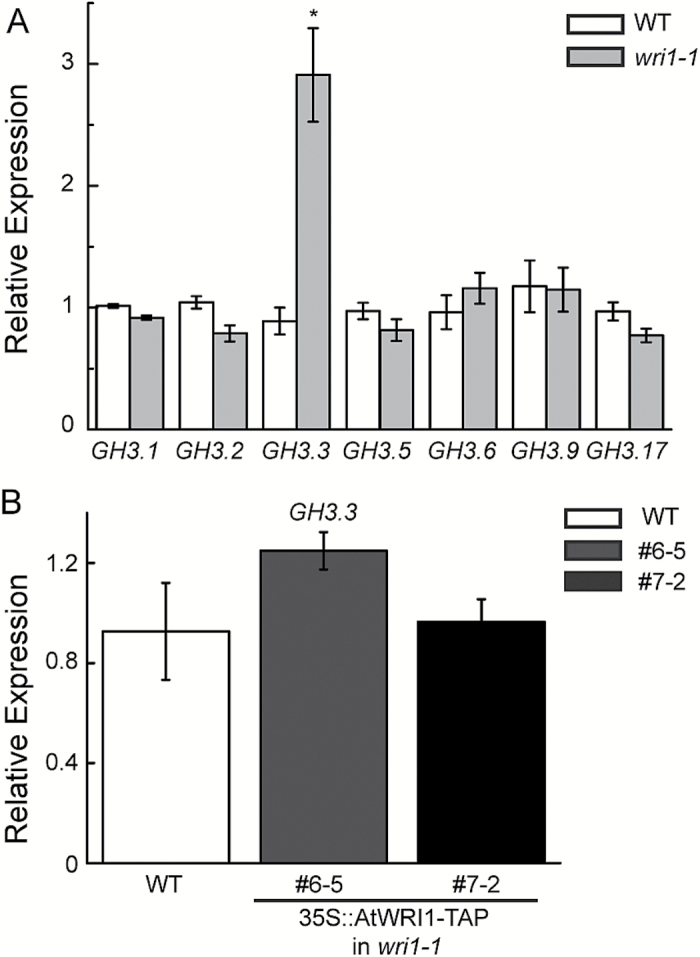
Expression analysis of the genes encoding indole-3 acetyl-aspartate synthetases (in the *GH3* gene family). (A) *GH3* transcripts of WT and *wri1-1* plants were measured by quantitative real-time PCR. Results are shown as means ±SE (*n*=3). * indicates a significant difference (*P*<0.05, *t*-test) compared with the WT. (B) *GH3.3* transcripts of the WT and transgenic *wri1-1* overexpressing *AtWRI1-TAP* (lines 6-5 and 7-2) were determined by quantitative real-time PCR. Results are shown as means ±SE (*n*=3).

We hypothesized that AtWRI1 might play a role in modulating the expression of genes involved in auxin signaling. To test this hypothesis, we surveyed the transcriptional profiles of roots of *wri1-1* and WT plants by microarray analysis. Notably, the expression of *GH3.3*, a member of the *GH3* gene family of auxin-responsive genes, was elevated in the mutant (Supplementary Table S2). To validate the microarray data, we determined the expression of *GH3.3* and other *GH3* genes by qRT-PCR. The expression of *GH3.3* was reproducibly elevated in *wri1-1* (Fig. 3A). Overexpressing *AtWRI1-TAP* in *wri1-1* reverted the increased expression of *GH3.3* in *wri1-1*. The expression of *GH3.3* in *AtWRI1-TAP* transgenic lines (#6-5 and #7-2) compared with WT plants was not significantly different (*P*>0.05, *t*-test) (Fig. 3B). This result indicates that the elevation of *GH3.3* expression in the *wri1-1* mutant is due to the mutation of *AtWRI1.* Expression of *GH3.15* was also elevated in *wri1-1* (Supplementary Fig. S3). However, expression of *GH3.10*, *GH3.12*, and *GH3.13* in *wri1-1* was reduced compared with the WT (Supplementary Fig. S3). In addition, we determined the expression of additional auxin-responsive genes of other families, including *IAA7*, *IAA17*, *IAA28*, *ARF6*, *ARF8*, and *ARF17*. Compared with WT plants, no obvious alteration was detected in the expression of the three *IAA* (Supplementary Fig. S4A) or *ARF* (Supplementary Fig. S4B) genes tested in the *wri1-1* mutant.

### AtWRI1 binds to the promoter of *GH3.3*

**Fig. 4.**
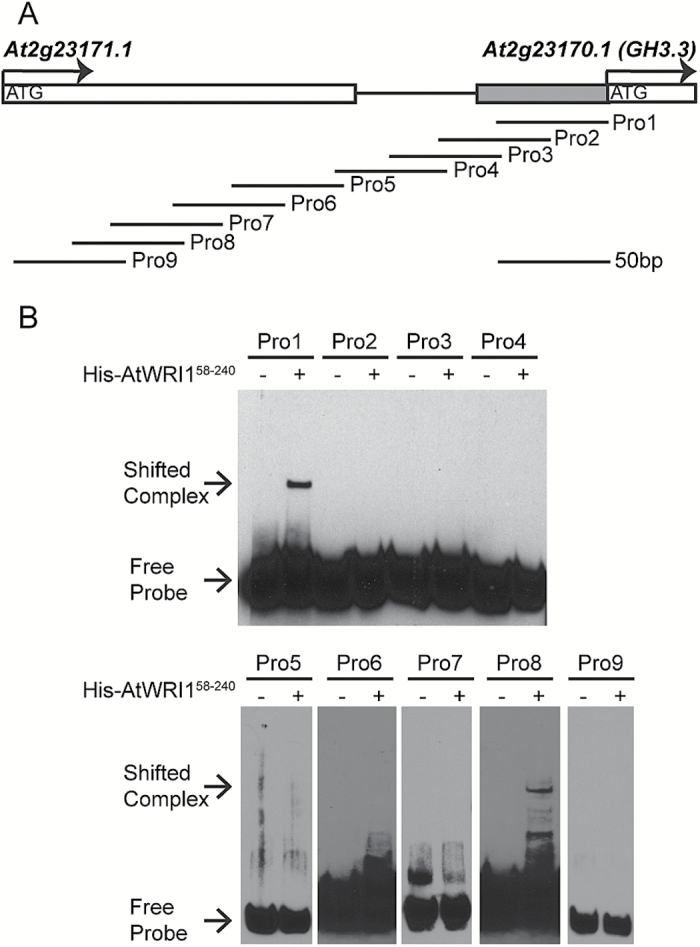
AtWRI1 binds to the promoter of *GH3.3*. (A) Schematic diagram of the promoter region of the *GH3.3* gene. A total of nine DNA probes have been designed from _*pro*_*GH3.3* for the EMSA. (B) Binding of the AP2 domain of AtWRI1 (amino acids 58–240) to various probes using EMSA.

To understand how AtWRI1 affects the expression of the *GH3.3* gene, we first performed bioinformatic analysis of the 1.0 kb promoter region of *GH3.3* (_*pro*_*GH3.3*). However, there was no recognizable AW-box in _*pro*_*GH3.3*. To examine whether the AtWRI1 protein can bind to _*pro*_*GH3.3*, we designed a series of DNA probes covering the region 268 bp upstream of the translational start site (Fig. 4A; Supplementary Fig. S5A). An EMSA indicated that the AtWRI1 protein was able to bind DNA probes Pro1 and Pro8 (Fig. 4B). Further alignment of the DNA sequences of Pro1 and Pro8 showed some similarity (Supplementary Fig. S5). Hence, they might contain the putative binding site(s) for AtWRI1 in _*pro*_*GH3.3*.

### Auxin sensitivity and transport are altered in the *wri1-1* mutant

We also examined the root elongation of WT and *wri1-1* seedlings in response to applied IAA. There was no apparent difference when seedlings were grown in the presence of high concentrations of IAA (10^–7^, 10^–6^, and 10^–5^ M). Both WT and *wri1-1* seedlings showed similar inhibition in response to IAA (Supplemenatry Fig. S6). When seedlings were grown on medium with relatively low concentrations of IAA (10^–9^ M and 10^–8^ M), *wri1-1* displayed more inhibition compared with the WT (Supplementary Fig. S6). This result suggests that *wri1-1* had a slightly higher sensitivity to applied IAA at least at some concentrations. This result was somewhat surprising because mutants with increased *GH3* gene expression and an elevated IAA-Asp level had been reported to display increased resistance to applied IAA (Nakazawa *et al.*, 2001; Staswick *et al.*, 2005; Park *et al.*, 2007). We hypothesized that mutation of *AtWRI1* might affect additional processes that mediate auxin homeostasis. Hence, we examined auxin transport in the WT and the *wri1-1* mutant. As shown in Supplementary Fig. S7, auxin transport was reduced in the *wri1-1* mutant. Compared with the WT, auxin transport was reduced 41% in roots of *wri1-1*.

### Expression of *PIN* genes is altered in the *wri1-1* mutant

**Fig. 5.**
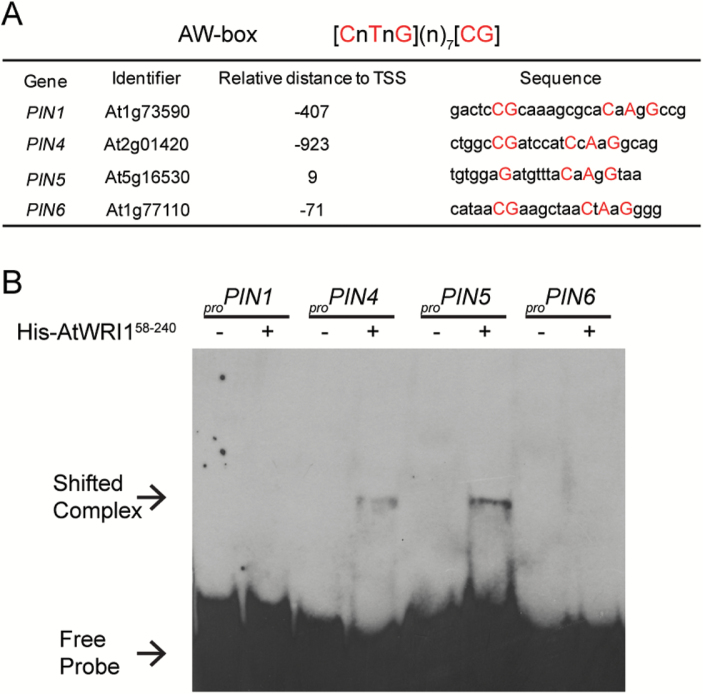
Examination of AtWRI1 binding to promoters of *PIN* genes. (A) *In silico* analysis of the AW-box in the promoter region of *PIN* genes. The AtWRI1-binding site AW-box was identified by AthaMap (http://www.athamap.de/index.php, last accessed 27 July 2017). The region from 1 kb upstream of the TSS (transcription start site) to 100 bp downstream of the TSS was screened for each gene. Nucleotides that match to the AW-box are shown with upper case letters. (B) Binding of the AP2 domain of AtWRI1 (amino acids 58–240) to the AW-box or AW-box-like sequence in the promoters of *PIN1*, *PIN4*, *PIN5*, and *PIN6*. The sequences of probes used in EMSA are listed in (A). (This figure is available in colour at *JXB* online.)

Previous studies have shown that expression of *PIN* genes is correlated with changes in auxin transport in Arabidopsis roots (Peer *et al.*, 2004). Thus, we hypothesized that the decreased auxin transport that we observed in the *wri1-1* mutant might be due to the altered expression of *PIN* genes. A qRT-PCR assay was performed, and the result indicated that the expression level of some *PIN* genes (*PIN1*, *PIN3*, *PIN5*, and *PIN6*) was reduced in *wri1-1* (Supplementary Fig. S8). In addition, we searched the presumed promoter regions (1 kb upstream of the transcription start site) of *PIN* genes for the AtWRI1 binding motif AW-box (Maeo *et al.*, 2009). An AW-box (or AW-box-like) motif was identified in the 1 kb sequences 5' of the transcriptional start of *PIN1*, *PIN4*, *PIN5*, and *PIN6* (Fig. 5A). Therefore, AtWRI1 might control the expression of *PIN* genes through binding to the AW-box motif. To test this hypothesis further, we performed EMSA experiments to probe whether AtWRI1 binds to _*pro*_*PIN* genes. The EMSA result indicates that AtWRI1 was able to bind to the AW-box within the context of the presumed _*pro*_*PIN4* and _*pro*_*PIN5* (Fig. 5B), but AtWRI1 did not appear to bind to the presumed _*pro*_*PIN1* and _*pro*_*PIN6* sequences (Fig. 5B).

## Discussion

Results presented above suggest that AtWRI1 plays a role in mediating auxin homeostasis by binding to the promoter of the auxin-responsive gene *GH3*, providing a molecular mechanism for how AtWRI1 might affect auxin homeostasis. It should be noted that AtWRI1 binds to _*pro*_*GH3.3* at a non-canonical motif. In addition, AtWRI1 might play a role in controlling the expression of auxin carrier genes by binding to the canonical AtWRI1 *cis*-element AW-box in the regulatory regions of select *PIN* genes.

### *GH3.3* is a genuine target gene of the transcription factor AtWRI1

Our data suggest that AtWRI1 is a transcriptional repressor for *GH3.3*. Previous studies have indicated that *GH* genes have potential roles in mediating auxin homeostasis and root development (Park *et al.*, 2007; Gutierrez *et al.*, 2012). Thus, aside from the well-established function of AtWRI1 in oil biosynthesis and the regulation of expression of fatty acid biosynthesis genes, AtWRI1 has a set of target genes probably unrelated to oil biosynthesis, which could cause potential side effects during the WRI1-based engineering of oil content.

A 50 bp DNA fragment in _*pro*_*GH3.3* is critical for AtWRI1 binding to _*pro*_*GH3.3* (Fig. 4). Further *in silico* analysis indicated that it is different from AuxREs or AW-box elements. Therefore, AtWRI1 binds to this *cis*-element in the auxin-responsive gene *GH3.3* in addition to its binding to the classical AtWRI1 AW-box-binding motif in genes related to fatty acid metabolism (Maeo *et al.*, 2009).

Expression of *GH3.3* was increased when the AtWRI1 protein was lost (*wri1-1*; Fig. 3A). In Arabidopsis, it has been suggested that *GH3.3*, *GH3.5*, and *GH3.6* have functional redundancy with regard to their effects on lateral root growth (Gutierrez *et al.*, 2012). We did not observe lateral root phenotypes in the *wri1-1* mutant (Fig. 1A). Thus, it was unclear whether the increased transcript of *GH3.3* in *wri1-1* led to the reduced root growth of the *wri1-1* plants. However, the endogenous level of IAA-Asp was elevated in *wri1-1* compared with the WT (Fig. 2B). *GH3.3* was suggested to be involved in auxin homeostasis including the biosynthesis of IAA-Asp (Gutierrez *et al.*, 2012). Therefore, we suggest that this might be the possible molecular mechanism explaining the reduced root growth that we observed in *wri1-1* plants.

In addition to the altered expression of *GH3.3*, *wri1-1* also had changes in the expression of other *GH3* genes such as *GH3.10*, *GH3.12*, *GH3.13*, and *GH3.15* (Supplementary Fig. S3). *GH3.10* antisense transgenic plants show primary root growth inhibition only under certain light conditions (Takase *et al.*, 2003). The roles of *GH3.12*, *GH3.13*, and *GH3.15* in root growth and auxin homeostasis still remain unknown at present. Therefore, we cannot rule out that the alterations of the expression of these *GH3* genes also result in modulating auxin homeostasis, which in turn affects the primary root growth of *wri1-1* plants.

### *PIN* genes might be another set of AtWRI1 target genes

Plant transcription factors such as XAL2, IDD14/15/16, and MYB88 have been shown to play an important role in controlling the expression of *PIN* genes (Cui *et al.*, 2013; Garay-Arroyo *et al.*, 2013; Wang *et al.*, 2015). Here, our data suggest that AtWRI1 might also be a transcription factor mediating the expression of *PIN* genes. Expression of four *PIN* genes was reduced in *wri1-1* compared with the WT (Supplementary Fig. S8), which might explain why *wri1-1* displayed reduced auxin transport in roots. *In silico* analysis (Fig. 5A) revealed that the AW-box (or AW-box like) motif was found in the promoter regions of three *PIN* genes (*PIN1*, *PIN5*, and *PIN6*) also showing reduced expression in *wri1-1* plants (Supplementary Fig. S8), which suggests that *PIN1*, *PIN5*, and *PIN6* might be AtWRI1 target genes. Although the AW-box in _*pro*_*PIN6* matches the AW-box identified previously (Maeo *et al.*, 2009), we did not detect the AtWRI1 binding to the AW-box in _*pro*_*PIN6* in the EMSA (Fig. 5B). It has been suggested that there is a 7 bp random sequence within the AW-box (Maeo *et al.*, 2009). Thus, the difference of a 7 bp random sequence between _*pro*_*PIN6* and promoters of AtWRI1 classical target genes (e.g. *BCCP2*) may cause the altered binding affinity of AtWRI1 in this case. Regarding the AW-box-like motif in _*pro*_*PIN1*, it contains a 9 bp random sequence instead of 7 bp, which might explain also why AtWRI1 did not bind to _*pro*_*PIN1* (Fig. 5B). Although AtWRI1 bound to the AW-box in the presumed _*pro*_*PIN4* (Fig. 5B), it should be noted that the AW-box-like motif in the presumed _*pro*_*PIN4* was located ~1 kb upstream from the transcriptional start site (Fig. 5A) and probably too far away to be directly involved in the regulation of PIN4 expression. Previously identified AW-boxes in promoter regions of AtWRI1 target genes are usually 300 bp upstream from the transcriptional start site (Maeo *et al.*, 2009), which might explain why expression of *PIN4* was not altered in the *wri1-1* mutant compared with the WT (Supplementary Fig. S8). In addition, imaging of _*pro*_*PIN4::PIN4-GFP* or immunolocalization of PIN4 protein in roots of *wri1-1* plants might need to be pursued in the future to clarify the effect of WRI1’s mutation on the expression of *PIN4*. There is no AW-box in the presumed _*pro*_*PIN3*, so AtWRI1 might bind to this promoter through an as yet unknown *cis*-element. In addition, a previous study indicated that PIN5 plays a role in mediating auxin homeostasis. Induction of *PIN5* expression in BY-2 cells results in reduced free IAA and increased IAA-Asp contents (Mravec *et al.*, 2009). In line with that, the endogenous IAA-Asp content was increased in *wri1-1* compared with the WT (Fig. 2B), while expression of *PIN5* was reduced in *wri1-1* (Supplementary Fig. S8). Taken together, mutation of *AtWRI1* led to expression changes in the genes encoding both protein families related to auxin homeostasis, *GH3* and *PIN* genes, probably leading to altered IAA metabolite levels and IAA sensitivity, and ultimately the observed root growth defect in the *wri1-1* mutant. While we do not yet fully understand how the misregulation of these genes causes the observed growth phenotype, recognizing the mere fact that WRI1 affects hormone homeostasis is of importance for the engineering of crops for increased oil content using the WRI1 transcription factor in the future.

## Supplementary data

Supplementary data are available at *JXB* online.

Fig. S1. Expression profiles of *AtWRI1* in Arabidopsis root.

Fig. S2. Growth phenotype of the WT and *wri1-1* on pH indicator plates.

Fig. S3. Gene expression of *GH3* genes of unknown function.

Fig. S4. Gene expression of *IAA* and *ARF* auxin- responsive genes.

Fig. S5. Probes for the promoter of *GH3.3* used in EMSA.

Fig. S6. Primary root growth inhibition in response to different concentrations of IAA.

Fig. S7. Auxin transport in roots of the WT and *wri1-1*.

Fig. S8. Expression of *PIN* genes.

Table S1. Quantitative real-time PCR primers used in this study.

Table S2. Genes expressed differently in roots of *wri1-1* compared with WT plants in microarray analysis.

## Supplementary Material

supplementary_figures_and_tables
