# Supplementary material for: The Arabidopsis WRINKLED1 transcription factor affects auxin homeostasis in roots
Source: J Exp Bot. 2017 Aug 26;68(16):4627–34. doi: 10.1093/jxb/erx275 (PMC5853644; doi:10.1093/jxb/erx275)
Supplement: supplementary_figures_and_tables [file erx275_suppl_supplementary_figures_and_tables.pdf]

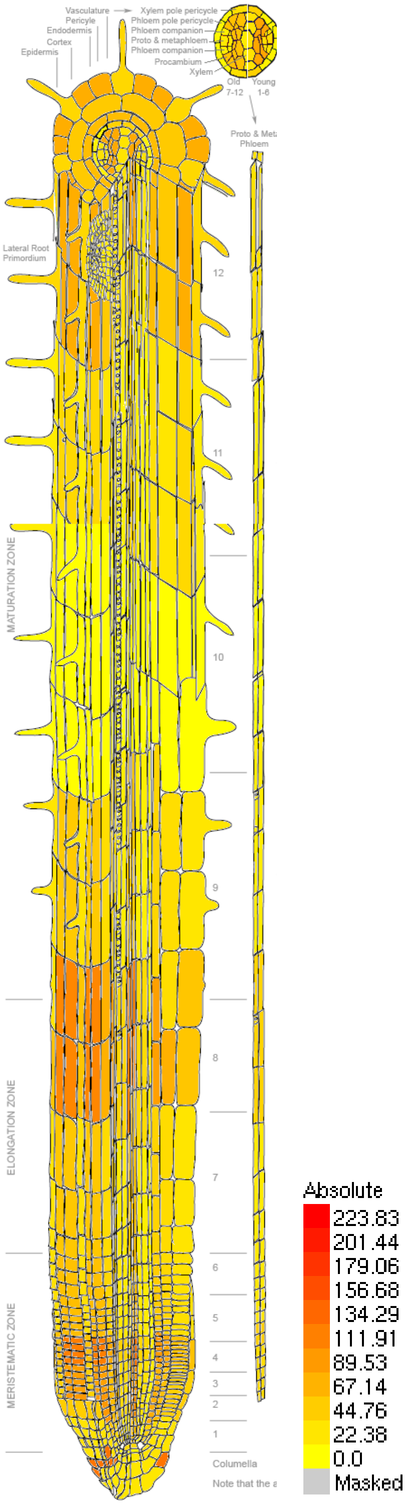

Figure S1. Expression profiles of *AtWRI1* in Arabidopsis root. Data are obtained from the Arabidopsis eFP browser (http://bar.utoronto.ca/efp/cgi-bin/efpWeb.cgi).

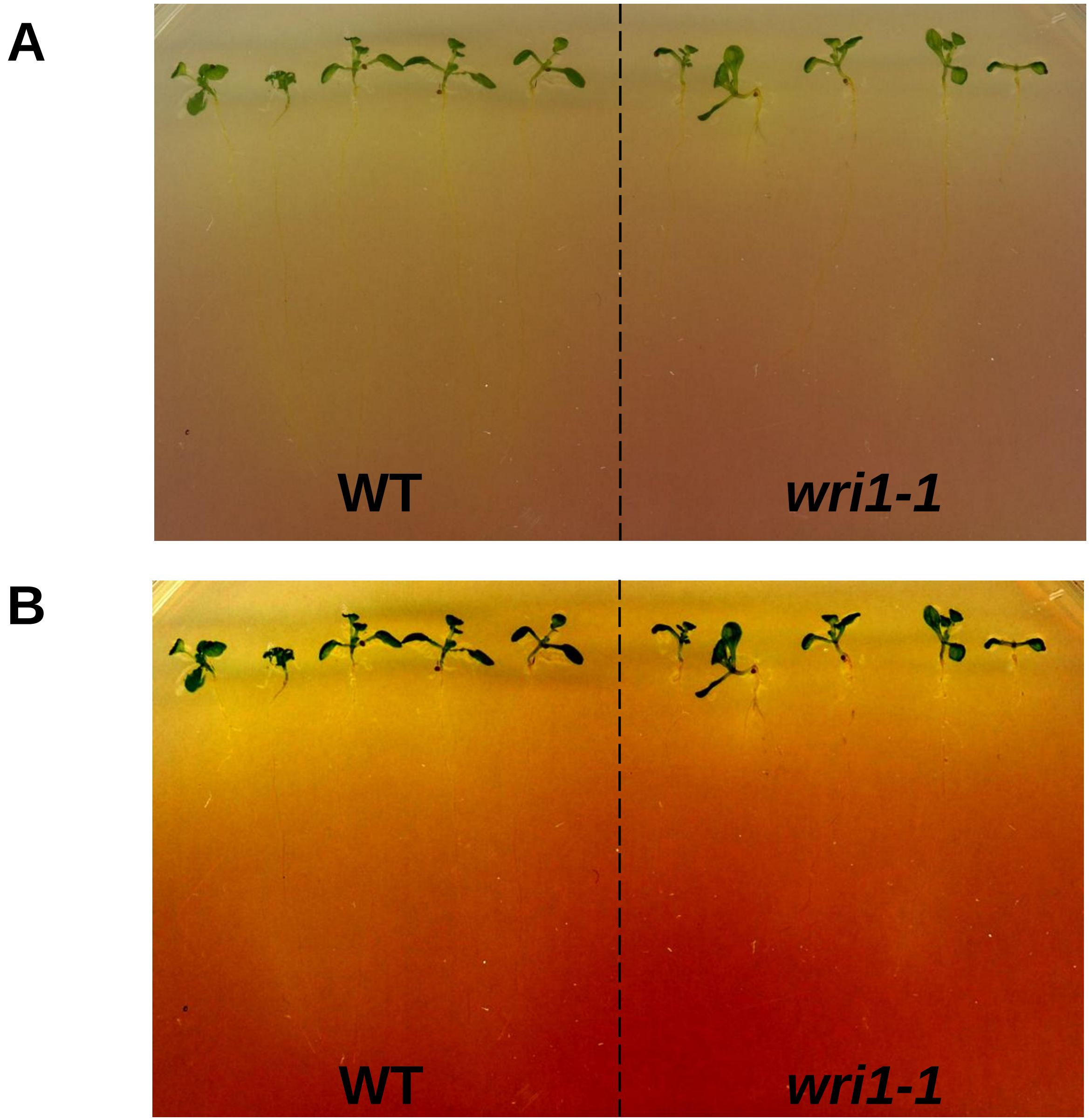

Figure S2. Growth phenotype of WT and *wri1-1* on pH indicator plates. **A**) Original picture of WT and *wri1-1* seedlings grown on pH indicator plates. **B**) Same picture as shown in **A**) with the contrast increased.

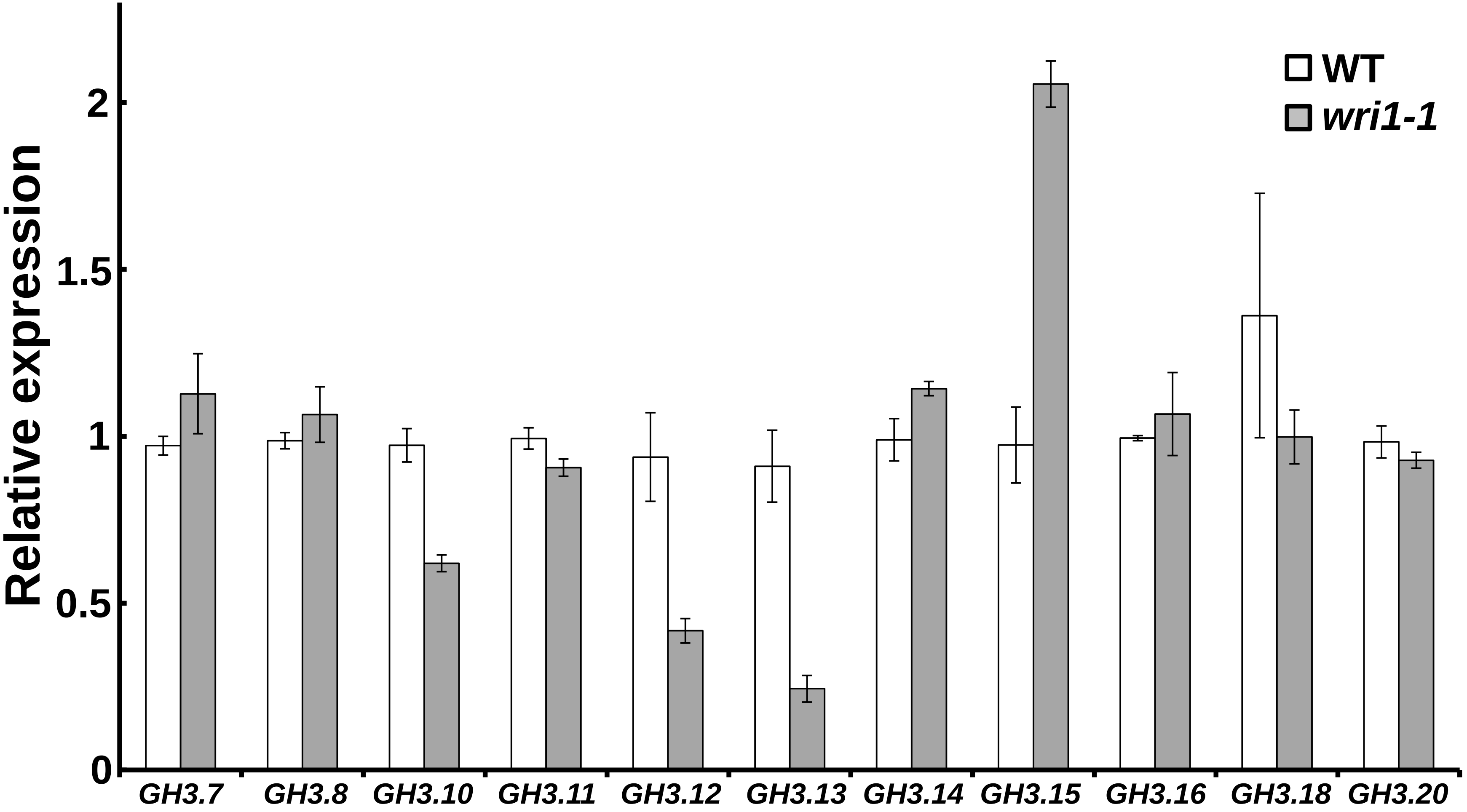

Figure S3. Gene expression of *GH3* genes of unknown function. *GH3* transcripts of WT and *wri1-1* plants were measured by quantitative real-time PCR. Results are shown as mean ± SE (n = 3).

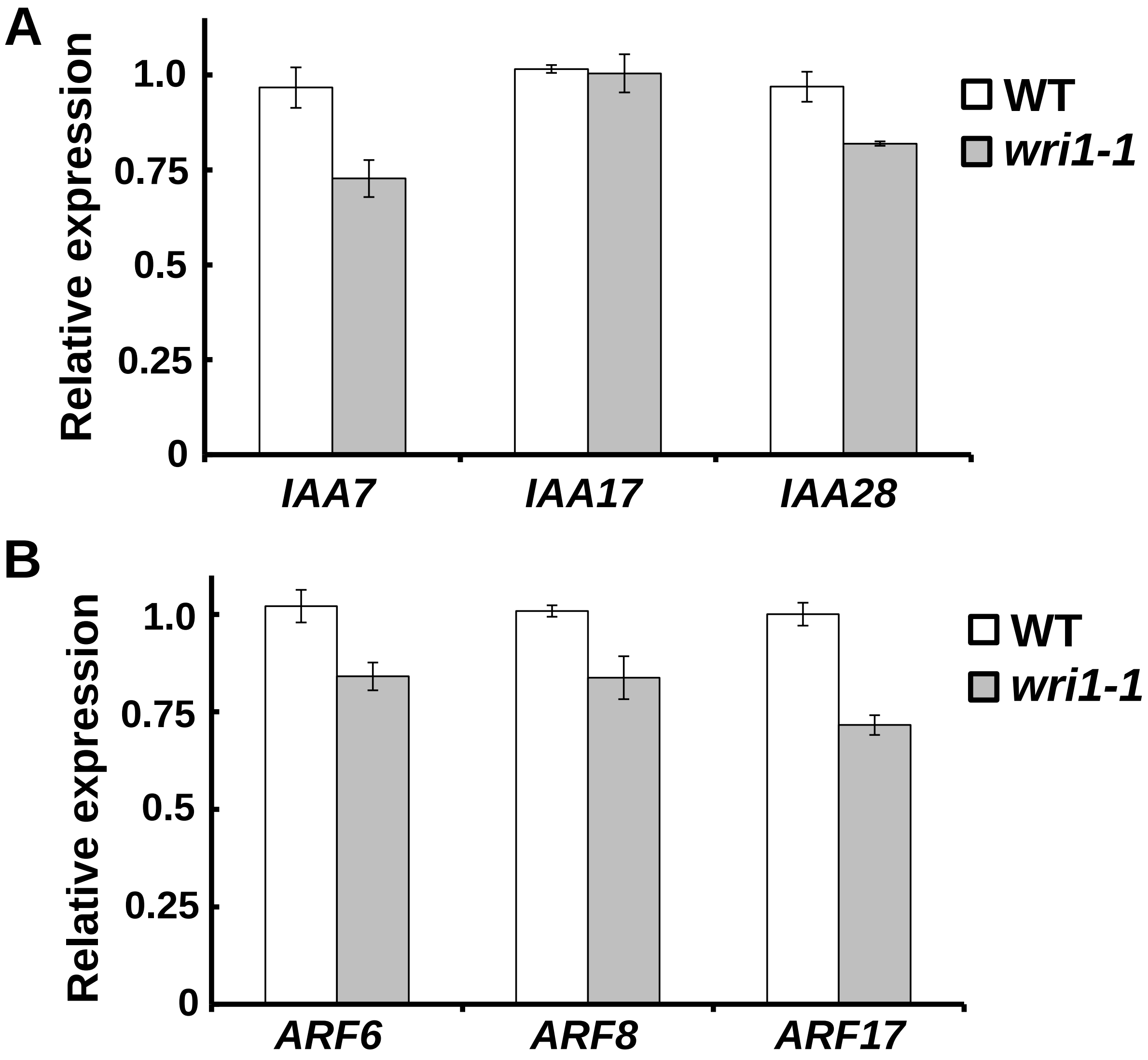

Figure S4. Gene expression of *IAA* and *ARF* auxin-responsive genes. **A**) *IAAs* and **B**) *ARFs* transcripts of WT and *wri1-1* plants were quantified by quantitative real-time PCR. Results are shown as mean ± SE (n = 3).

A

GH3.3 Pro1 GATTAAAATGGTATTTGTAAGTGATAATATTAGACTTTGTGTTTGATGAT
GH3.3 Pro2 AATATTAGACTTTGTGTTTGATGATGAGGAGATATTGAAACTCGACGAGT
GH3.3 Pro3 GAGGAGATATTGAAACTCGACGAGTATGGGGTTGTATTTATAGTGGCAAG
GH3.3 Pro4 ATGGGGTTGTATTTATAGTGGCAAGAAGGAGAAAACCAACGTCAGCCAAA
GH3.3 Pro5 AGCCAAATTCCACGTCGACATAAAAGTGGGCTCGGTTTGTTATCGTTAGA
GH3.3 Pro6 TGGGCTCGGTTTGTTATCGTTAGACCGAGACAGGGGACAAGAGGGACCGC
GH3.3 Pro7 CCGAGACAGGGGACAAGAGGGACCGCGGTCACGTAATCTTTGGCTAGTTT
GH3.3 Pro8 AGGGACCGCGGTCACGTAATCTTTGGCTAGTTTTGGGCAGACATGTGGGACTGA
GH3.3 Pro9 TGGGCAGACATGTGGGACTGATATGTCTGCGCCACGTTGGCATGTTACTT

B

```
Pro1      gattaaaatggtatttgtaagtgataatattagactttgtgtttgatgat------------
Pro8      --------agggaccgcggtcacgtaatctttggctagttttgggcagacatgtgggactga
                   ** *           **** ** * **   * *  *  **
```

Figure S5. Probes for the promoter of *GH3.3* used in EMSA. **A**) The DNA sequences of probes used for the EMSA are shown. **B**) Alignment of the sequences of pro1 and pro8 by CLUSTAL O program (http://www.ebi.ac.uk/Tools/msa/clustalo/).

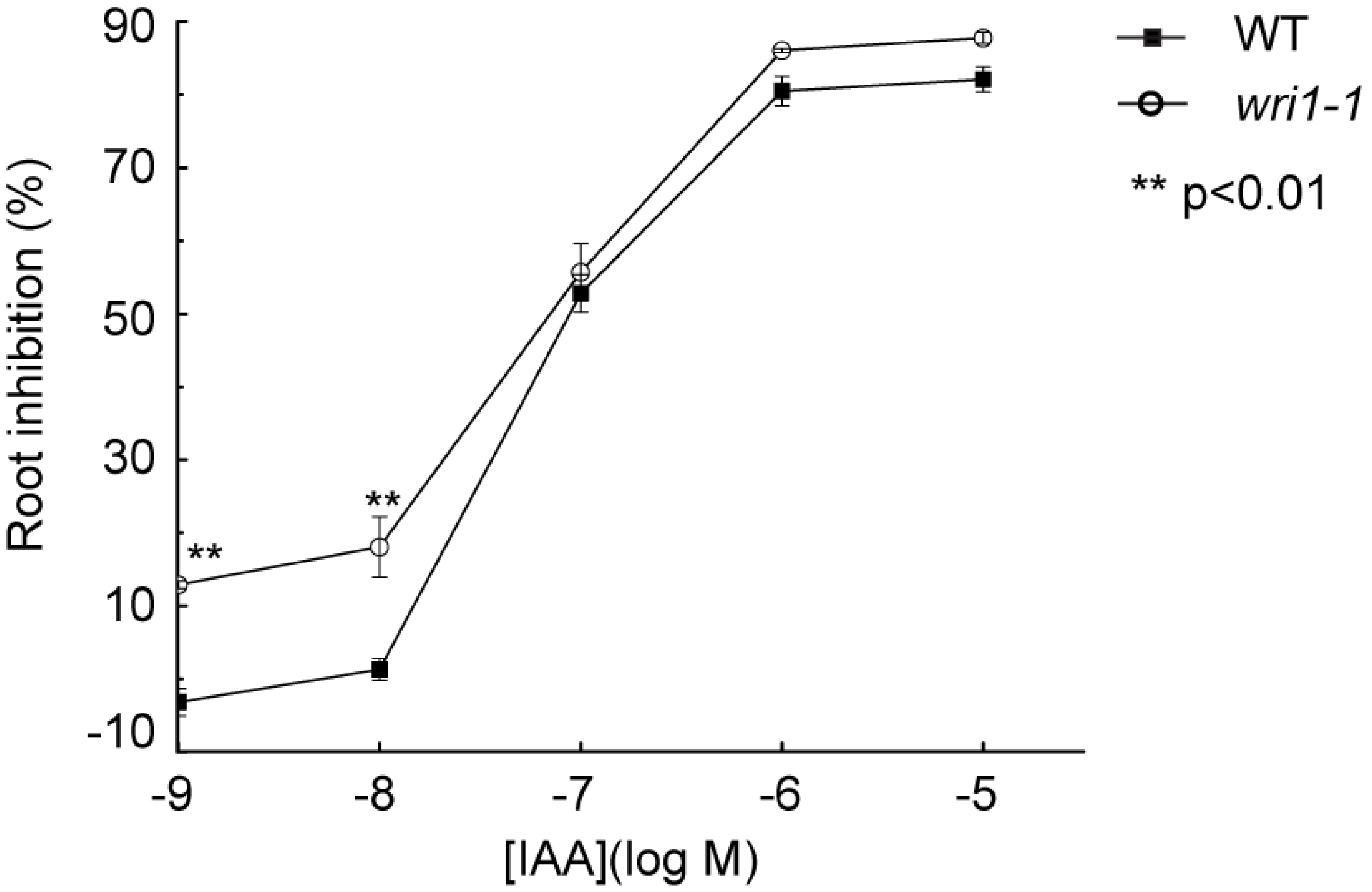

Figure S6. Primary root growth inhibition in response to different concentrations of IAA. 4-day-old seedlings of WT and *wri1-1* were transferred to growth media with various concentrations of IAA and continued growing for 6 days. In each experiment, 13 seedlings were measured . Results shown are based on three independent experiments as mean ± SE (n=3). "**"indicate significant difference (P<0.01, t-test) to WT.

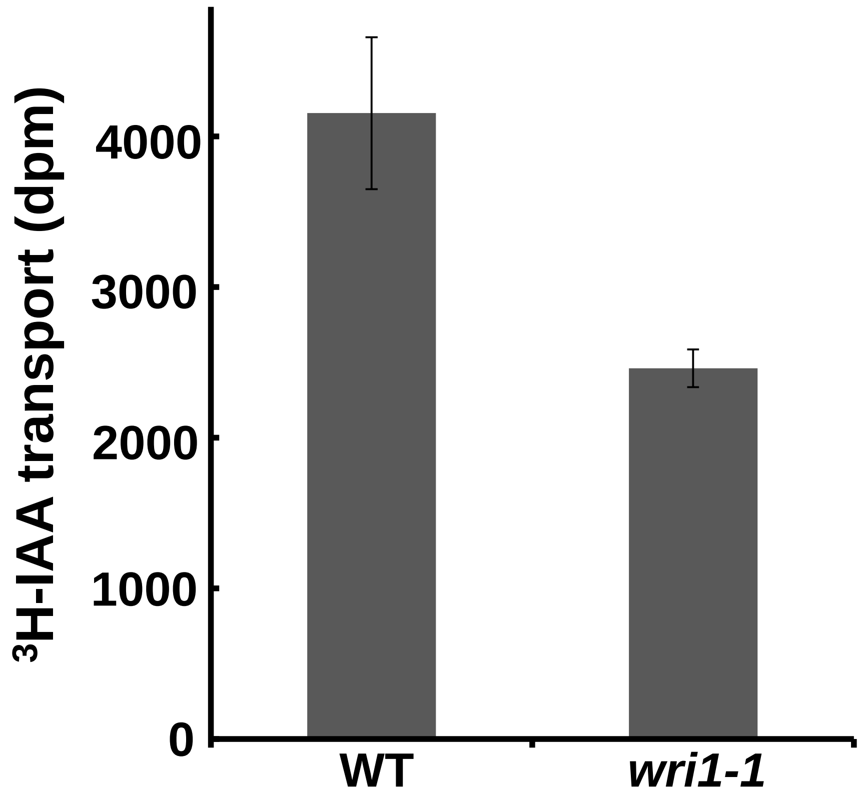

Figure S7. Auxin transport in roots of WT and *wri1-1*. Values are means ± SE (n=3).

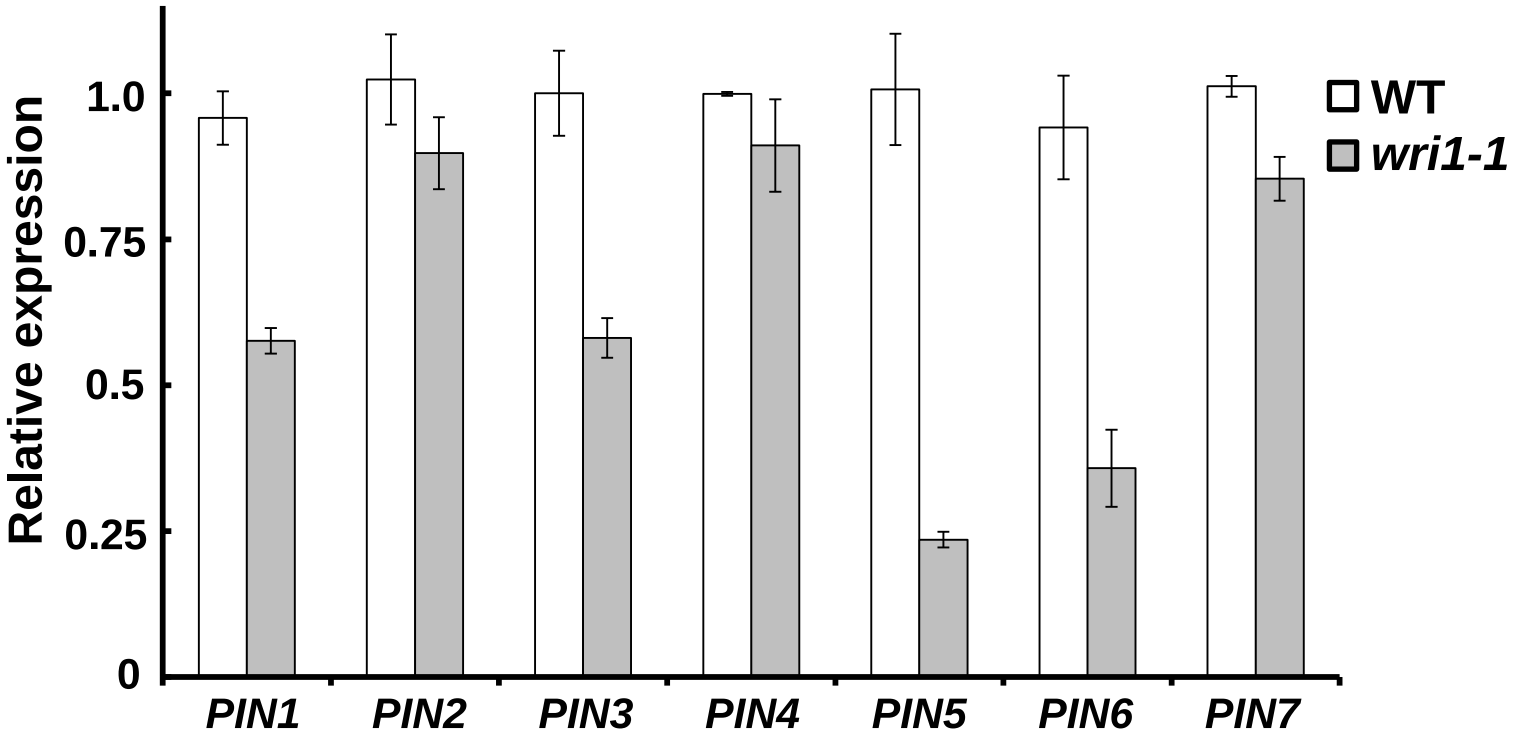

Figure S8. Expression of *PIN* genes. *PINs* transcripts of WT and *wri1-1* plants were quantified by quantitative real-time PCR. Results are shown as mean ± SE (n = 3-4).

Primer Name	Sequence 5' to 3'
GH3.1-FW	GTCCTCCTCAGCATCGACTC
GH3.1-RV	ATCGTGCTTGTGTCTGCGTA
GH3.2-FW	GCTGTTTGGAGATGGAGGAA
GH3.2-RV	CCGATCGATTTATCAGCGAC
GH3.3-FW	ATCAGTACAAGGTGCCGAGG
GH3.3-RV	AAAGCTGGGCTGAAGTGTGT
GH3.4-FW	TGGAGGAATCGCTGAACGCGG
GH3.4-RV	TGCCGTTCTGCACCACACGTA
GH3.5-FW	TACTTCAGCCCCAAGTGTCC
GH3.5-RV	TCTTCACGTTCCACATTCCA
GH3.6-FW	CTTGAGAAACATTGGCCTGAG
GH3.6-RV	CAGATTCGACGAAATCAGCA
GH3.7-FW	TGATCTGTTTAAGGCGGTGA
GH3.7-RV	TAGTGTCCCGGGATAGTGGA
GH3.8-FW	GACCTTTTAAACGCGGTCAC
GH3.8-RV	GTCGTTGCGGTATTTTGCTT
GH3.9-FW	GCAGGACGAAAGAGAGATCG
GH3.9-RV	AACGACCCTCCTTGGCTTAT
GH3.10-FW	GGGAAATCAGAGGAGAAGCA
GH3.10-RV	AACGTTCCTCGTTCCACAAC
GH3.11-FW	GTTCATCGGCTGGACAGTTT
GH3.11-RV	TCAAAACGCTGTGCTGAAGT
GH3.12-FW	AGGGAGAGGAGAAGGAGACG
GH3.12-RV	ATCGATCCGTCTTTGAATCG
GH3.13-FW	GTGTGGATTCGTCCTCGTTT
GH3.13-RV	TCAACGGTGAAGCAACATTC
GH3.14-FW	CACTAGCCGTGTGGATTCCT
GH3.14-RV	GCAGCATTCCTCCAAAACAT
GH3.15-FW	CCGCGATGTTATGGAAGAAT
GH3.15-RV	CGCCAGGCTTAACAACCTTA
GH3.16-FW	GTTAAACCTGGCGCTTTTGA
GH3.16-RV	AGGCTTCTTCATGCGTCACT

GH3.17-FW	TCGTCTCTGCTCAGCCCATCACA
GH3.17-RV	GCTGTAGAAGGCATCAACTTCGGCT
GH3.18-FW	ATCTCCCAAGGAGGTTCGAT
GH3.18-RV	TTTCGCTAAAGAATCGAGCAA
GH3.19-FW	TGGTTCGGTTGCTCAATACA
GH3.19-RV	GAGGAGGAGATTTTTGGCTGT
GH3.20-FW	TTGGGGAAGGAAAAGTGATG
GH3.20-RV	GATGGCCGGTTCTTGAAATA
IAA7-FW	GCCAAGCTACGAGGACAAAG
IAA7-RV	CTCTCGGAGCAAGTCCAACT
IAA17-FW	AAGACGGTGATTGGATGCTC
IAA17-RV	TCTGCTCTTGCACTTCTCCA
IAA28-FW	ATGCCGTTGACCAACTCTTC
IAA28-RV	CATCCCCGACCAGAACTTTA
ARF6-FW	AATGGGAAAAAGAGGCCTTG
ARF6-RV	ATGCGATACCGTTACCGAGA
ARF8-FW	GACCCATGGGAGTCATTTGT
ARF8-RV	TAGAGATGGGTCGGGTTTTG
ARF17-FW	GGAAATGACCTGGTTGGAAA
ARF17-RV	GACTCTTTGCTGCCATCCTC
PP2A-FW	TAACGTGGCCAAAATGATGC
PP2A-RV	GTTCTCCACAACCGCTTGGT
PIN1-FW	GGCATGGCTATGTTCAGTCTTGGG
PIN1-RV	ACGGCAGGTCCAACGACAAATC
PIN2-FW	TCACGACAACCTCGCTACTAAAGC
PIN2-RV	TGCCCATGTAAGGTGACTTTCCC
PIN3-FW	AAGGCGGAAGATCTGACCAAGG
PIN3-RV	TGCTGGATGAGCTACAGCTTTG
PIN4-FW	ACAACGTGGCAACGGAACAATC
PIN4-RV	GCCGATATCATCACCACCACTC
PIN5-FW	CCATCGGCTCTATTGTCCTTG
PIN5-RV	GCGACGAGCACAGGTAGAGA
PIN6-FW	TGGGCCGTTTTCTTCAAAGC
PIN6-RV	GATTGATCCGGCTGCTTGAC

PIN7-FW	CGTGTGGCCATTGTTCAAGCTG
PIN7-RV	CCCTGTACTCAAGATTGCGGGATG

Table S1. Quantitative real-time PCR primers used in this study.

Probe ID	AGI	fold change (WT/*wri1-1*)	P.Value	Annotation
256940_at	At3g30720	0.02	6.15E-12	QQS__qua-quine starch
265560_at	At2g05520	0.29	2.23E-06	ATGRP-3_ATGRP3_GRP-3_GRP3__glycine-rich protein 3
260522_x_at	At2g41730	0.34	4.34E-06	unknown protein; BEST Arabidopsis thaliana protein match is: unknown protein (TAIR:AT5G24640.1)
252347_at	At3g48130	0.37	2.73E-06	Ribosomal protein L13 homolog
254150_at	At4g24350	0.40	4.69E-06	Phosphorylase superfamily protein
245076_at	At2g23170	0.47	1.22E-05	GH3.3__Auxin-responsive GH3 family protein
267262_at	At2g22990	0.49	5.37E-06	SCPL8_SNG1__sinapoylglucose 1
265817_at	At2g18050	0.49	1.73E-05	HIS1-3__histone H1-3
251799_at	At3g55520	0.49	1.20E-06	FKBP-like peptidyl-prolyl cis-trans isomerase family protein
258727_at	At3g11930	0.50	4.23E-06	Adenine nucleotide alpha hydrolases-like superfamily protein
263948_at	At2g35980	0.52	1.06E-05	ATNHL10_NHL10_YLS9
263909_at	At2g36490	0.53	1.59E-05	DML1_ROS1__demeter-like 1
263515_at	At2g21640	0.59	1.16E-05	Encodes a protein of unknown function that is a marker for oxidative stress response.
263491_at	At2g42600	0.62	3.40E-05	ATPPC2_PPC2__phosphoenolpyruvate carboxylase 2
256502_at	At1g36730	0.67	3.97E-05	Translation initiation factor IF2/IF5
260392_at	At1g74030	1.54	2.80E-05	ENO1__enolase 1
257895_at	At3g16950	1.57	3.83E-05	LPD1_ptlpd1__lipoamide dehydrogenase 1
245220_at	At1g59171	1.58	2.84E-05	Inositol-pentakisphosphate 2-kinase family protein
249384_at	At5g39890	1.61	1.00E-05	Plant cysteine oxidase 2
254845_at	At4g11820	1.61	2.45E-05	HMGS_MVA1__hydroxymethylglutaryl-CoA synthase / HMG-CoA synthase / 3-hydroxy-3-methylglutaryl coenzyme A synthase
259969_at	At1g76550	1.62	2.47E-05	Phosphofructokinase family protein
262157_at	At1g52670	1.65	7.88E-06	BCCP-like protein1, BLP1
255558_at	At4g01900	1.65	4.00E-05	GLB1_PII__GLNB1 homolog
256160_at	At1g30120	1.69	7.58E-06	PDH-E1 BETA__pyruvate dehydrogenase E1 beta
259095_at	At3g05020	1.70	1.68E-05	ACP_ACP1__acyl carrier protein 1
266426_x_at	At2g07140	1.71	5.92E-06	F-box and associated interaction domains-containing protein
266426_x_at	At3g44120	1.71	5.92E-06	Phosphoenolpyruvate/phosphate translocator

249366_at	At5g40610	1.72	2.42E-06	NAD-dependent glycerol-3-phosphate dehydrogenase family protein
258086_at	At3g25860	1.73	6.88E-06	LTA2_PLE2__2-oxoacid dehydrogenases acyltransferase family protein
258485_at	At3g02630	1.80	1.02E-05	Plant stearoyl-acyl-carrier-protein desaturase family protein
264419_s_at	At1g43310	1.80	1.42E-05	Nucleotide-sugar transporter family protein
264419_s_at	At5g33320	1.80	1.42E-05	Phosphoenolpyruvate/phosphate translocator
247345_at	At5g63760	1.88	1.51E-06	ARI15_ATARI15__RING/U-box superfamily protein
248903_at	At5g46290	1.89	1.54E-05	KAS I_KAS1__3-ketoacyl-acyl carrier protein synthase I
248690_at	At5g48230	1.90	2.65E-05	ACAT2_EMB1276__acetoacetyl-CoA thiolase 2
266035_at	At2g05990	1.93	2.14E-06	ENR1_MOD1__enoyl-ACP reductase
246565_at	At5g15530	1.95	4.07E-06	BCCP2_CAC1-B__biotin carboxyl carrier protein 2
249490_s_at	At5g39110 At5g39120 At5g39150 At5g39180	2.06	2.16E-05	RmlC-like cupins superfamily protein
260982_at	At1g53520	2.08	1.62E-06	Fatty acid binding protein
265843_at	At2g35690	2.11	9.09E-07	ACX5__acyl-CoA oxidase 5
252863_at	At4g39800	2.11	1.17E-06	ATIPS1_ATMIPS1_MI-1-P SYNTHASE_MIPS1__myo-inositol-1-phosphate synthase 1
253660_at	At4g30140	2.17	2.09E-05	CDEF1__GDSL-like Lipase/Acylhydrolase superfamily protein
249474_s_at	At5g39130 At5g39160 At5g39190	2.19	1.97E-05	RmlC-like cupins superfamily protein
251378_at	At3g60660	2.42	1.12E-07	unknown protein; CONTAINS InterPro DOMAIN/s: Protein of unknown function DUF1395 (InterPro:IPR009829)
250472_at	At5g10210	2.43	1.99E-05	CONTAINS InterPro DOMAIN/s: C2 calcium-dependent membrane targeting (InterPro:IPR000008); BEST Arabidopsis thaliana protein match is: unknown protein (TAIR:AT5G65030.1); Has 1807 Blast hits to 1807 proteins in 277 species: Archae - 0; Bacteria - 0; Metazoa - 736; Fungi - 347; Plants - 385; Viruses - 0; Other Eukaryotes - 339 (source: NCBI BLink).
261335_at	At1g44800	2.43	3.45E-05	nodulin MtN21 /EamA-like transporter family protein

259813_at	At1g49860	2.44	1.65E-05	ATGSTF14_GSTF14__glutathione S-transferase (class phi) 14
260475_at	At1g11080	2.47	3.50E-05	scpl31__serine carboxypeptidase-like 31
264014_at	At2g21210	2.65	2.43E-05	SAUR-like auxin-responsive protein family
249675_at	At5g35940	2.88	5.49E-07	Mannose-binding lectin superfamily protein
267626_at	At2g42250	3.27	5.72E-06	CYP712A1__cytochrome P450, family 712, subfamily A, polypeptide 1
245571_at	At4g14695	3.47	7.79E-07	Uncharacterised protein family (UPF0041)

Table S2. Genes expressed differently in roots of *wri1-1* compared to WT plants in microarray analysis.
